# Arginine deprivation: a potential therapeutic for cancer cell metastasis? A review

**DOI:** 10.1186/s12935-020-01232-9

**Published:** 2020-05-06

**Authors:** Houssam Al-Koussa, Nour El Mais, Hiba Maalouf, Ralph Abi-Habib, Mirvat El-Sibai

**Affiliations:** Department of Natural Sciences, School of Arts and Sciences, Lebanese American University, P.O. Box: 13-5053, Chouran, 1102 2801 Beirut, Lebanon

**Keywords:** Arginine, Arginase, Cell migration, Rho-GTPases

## Abstract

Arginine is a semi essential amino acid that is used in protein biosynthesis. It can be obtained from daily food intake or synthesized in the body through the urea cycle using l-citrulline as a substrate. Arginine has a versatile role in the body because it helps in cell division, wound healing, ammonia disposal, immune system, and hormone biosynthesis. It is noteworthy that l-arginine is the precursor for the biosynthesis of nitric oxide (NO) and polyamines. In the case of cancer cells, arginine de novo synthesis is not enough to compensate for their high nutritional needs, forcing them to rely on extracellular supply of arginine. In this review, we will go through the importance of arginine deprivation as a novel targeting therapy by discussing the different arginine deprivation agents and their mechanism of action. We will also focus on the factors that affect cell migration and on the influence of arginine on metastases through polyamine and NO.

## Background

Cancer is associated with high mortality rates although significant improvements have been made in early detection and treatment [1, 2]. The most potent and conventional approach involves the usage of chemotherapy, radiotherapy, and surgery depending on the type and stage of the tumor [3, 4]. However, such cytotoxic and nonselective approaches could lead to severe damage to normal cells [1]. This opened the door for more selective approaches, including stem cell therapy, hormone therapy, immunotherapy and amino acid deprivation therapy [5–8]. The latter depends on the fact that cancer cells proliferate at a much higher rate than normal cells, requiring higher amounts of nutrients and exceeding their own capability to synthesize amino acids [9, 10]. Therefore, nonessential amino acids become essential, and the cancer cell becomes auxotrophic to them [11, 12]. This method is even more effective when the normal metabolism of a certain amino acid is disrupted in the cancer cell, making the cell more dependent on the environment and thus making the therapy more selective and efficient [13].

One of the promising amino acid deprivation therapies is arginine deprivation, which is based on introducing an arginine-depleting agent. Arginine is a semi essential amino acid; it is taken from dietary sources and synthesized through the urea cycle in the kidneys. Some types of cancer cells require arginine for growth, making them either arginine auxotrophic or partially auxotrophic. Interestingly, different studies showed that cancer cell migration has been affected by the depletion of arginine. Although motility is a normal physiological process associated with wound healing, embryonic development, and immune responses, it is a process used by cancer cells to metastasize to other organs [14, 15]. Therefore, the depletion of arginine impairs the cancer cell’s ability to metastasize. However, the mechanism of impairment is still under investigation.

In this review, we will briefly discuss how arginine deprivation could be used as a targeting therapeutic for both arginine auxotrophic and partially auxotrophic types of cancer. Moreover, we will give examples of the different types of arginine deprivation agents and their mechanism of action. Then, we will introduce the role of arginine deprivation in effecting cell migration through NO and polyamines. Finally, we will discuss other factors coordinating motility along with the role of RhoA, Rac1, and Cdc42 in invasion, focal adhesions formation and cell motility.

### Arginine

Arginine is considered a non-essential amino acid since the cell is capable of synthesizing its own arginine. However, when it is under catabolic stress, it relies heavily on acquiring arginine from the environment, making arginine “conditionally” essential [13, 16]. The synthesis of arginine is done through the urea cycle, which normally takes place in the kidney. The de novo synthesis of arginine includes the conversion of l-citrulline, by arginosuccinate synthetase (ASS1) and arginosuccinate lyase (ASL), to arginine [13]. Consequently, the addition of l-citrulline to arginine deprived cancer cells could rescue the cells and increase the flux of its urea cycle [17–19]. This is accomplished by the ASS1 enzyme that catalyzes the conversion of l-citrulline and aspartic acid to arginosuccinate, which is then converted by ASL to l-arginine and fumaric acid. l-ornithine could also be a substrate to produce arginine through its conversion by ornithine transcarbamylase (OCT). Some tumors have their arginine metabolizing enzymes downregulated, preventing them from producing arginine from available substrates and making them arginine auxotrophic [20–22].

### Arginine depriving agents

One of the first arginine deprivation agents to be synthesized was arginine deaminase (ADI), which metabolizes arginine, preventing cells in culture from growing [23]. However, it had limitations such as it is only effective on cancer cells lacking the ASS1 enzyme, it has low serum half-life, and it is highly immunogenic [24, 25]. To address such problems, polyethylene glycol (PEG) was attached to it, and it was given in high doses to increase efficiency [13, 23].

Synthesized human Arginase 1 (HuArgI) is another arginine deprivation agent that was used to target arginine in auxotrophic cancer cell lines. However, due to its limitations, it was enhanced by adding PEG and substituting Mn^2+^ with Co^2+^ to become HuArgI (Co)-PEG5000, which lasts longer in serum, has an improved catalytic activity and is less exposed to the immune system [17, 26, 27].

### Implication of arginine deprivation on cancer therapy

If the cells were not rescued upon adding l-citrulline after arginine depletion, then the cells exhibit arginine auxotroph. For example, l-citrulline failed to rescue ovarian cancer cell lines, SKOV3 and PA1, from cytotoxicity after arginine deprivation. Thus, those cell lines are completely auxotrophic to arginine. However, CaOV3, a third ovarian cancer cell line, was rescued upon the addition of l-citrulline, making this cell line a partially auxotrophic one [17]. Similarly, in the case of pancreatic cancer cells some cell lines were rescued when l-citrulline was added to them while others were not [17, 28]. Moreover, in the case of colorectal cancer, all the tested cell lines, including Caco-2, were rescued when adding l-citrulline, making them partial auxotrophic [29].

Depriving cancer cells from arginine does not only have a cytotoxic effect but also induces cell cycle arrest in some types of cancer cells. Cell cycle analysis was done on the surviving population of ovarian and pancreatic cancer cells to examine the effect of arginine deprivation on the cell cycle. Upon the addition of HuArgI (Co)-PEG5000 to several ovarian and pancreatic cancer cell lines, results showed that there was a cell cycle arrest at G0/G1 after 48 h and 72 h of treatment, respectively [17, 28]. Results also demonstrated a decrease in the number of cells present at the S and G2 phase, showing that arginine caused cell cycle arrest to the surviving ovarian cancer cells [17].

Different types of tumors undergo different mechanisms of cell death when deprived from arginine. Some tumors like acute lymphoblastic leukemia (ALL) and hepatocellular carcinoma exhibit cell death by apoptosis while other types of cancer cells like ovarian, pancreatic, acute myeloid leukemia (AML), and glioblastoma multiforme (GBM) showed a caspase-independent, non-apoptotic cell death [17, 18, 28, 30]. The latter is a process known as autophagy, which is when the cell degrades itself to form missing nutrients in response to starvation. Thus, this process is the first protective mechanism against cell death, yet on the long term it results in non-apoptotic death [17, 19, 31]. Chloroquine (CQ), an autophagy inhibitor, was added to ovarian, pancreatic, AML, and GBM cells when treated with HuArgI (CO) PEG-5000. As expected, autophagy was inhibited and the drug’s cytotoxicity decreased, indicating that the cells were dying after treatment by autophagy. Moreover, there were no caspase activation and no loss in the membrane integrity, proving that cell death was not due to apoptosis [17, 28].

### The effects of arginine deprivation on cell migration and invasion

After highlighting the role of arginine depletion on cell viability, it is important to look at its effect on cell migration. The enzyme ASS1 is important in the synthesis of arginine de novo by the cell. The absence or presence of ASS1 differentiate the cancer cell lines into arginine complete auxotrophic or partial auxotrophic, respectively [17, 18, 28, 30]. A previous study proved that ASS1 is expressed in human gastric cancer cell lines and its suppression efficiently hinders gastric cancer cell lines metastatic capabilities in vitro and in vivo. Moreover, this study indicated that when the levels of arginine drops, and it is accompanied with silencing the expression of ASS1, gastric cancer cell lines migration capability got hindered [32]. In another study, a western blot was performed on colon cancer Caco-2 cell line, showing a decrease in the level of ASS1 expression after the cells were deprived from arginine by HuArgI (Co)-PEG 5000. However, the levels of ASS1 expression were restored with the addition of l-citrulline [29]. This indicates that ASS1 expression and arginine concentration play a crucial role in cancer metastasis.

**Fig. 1:**
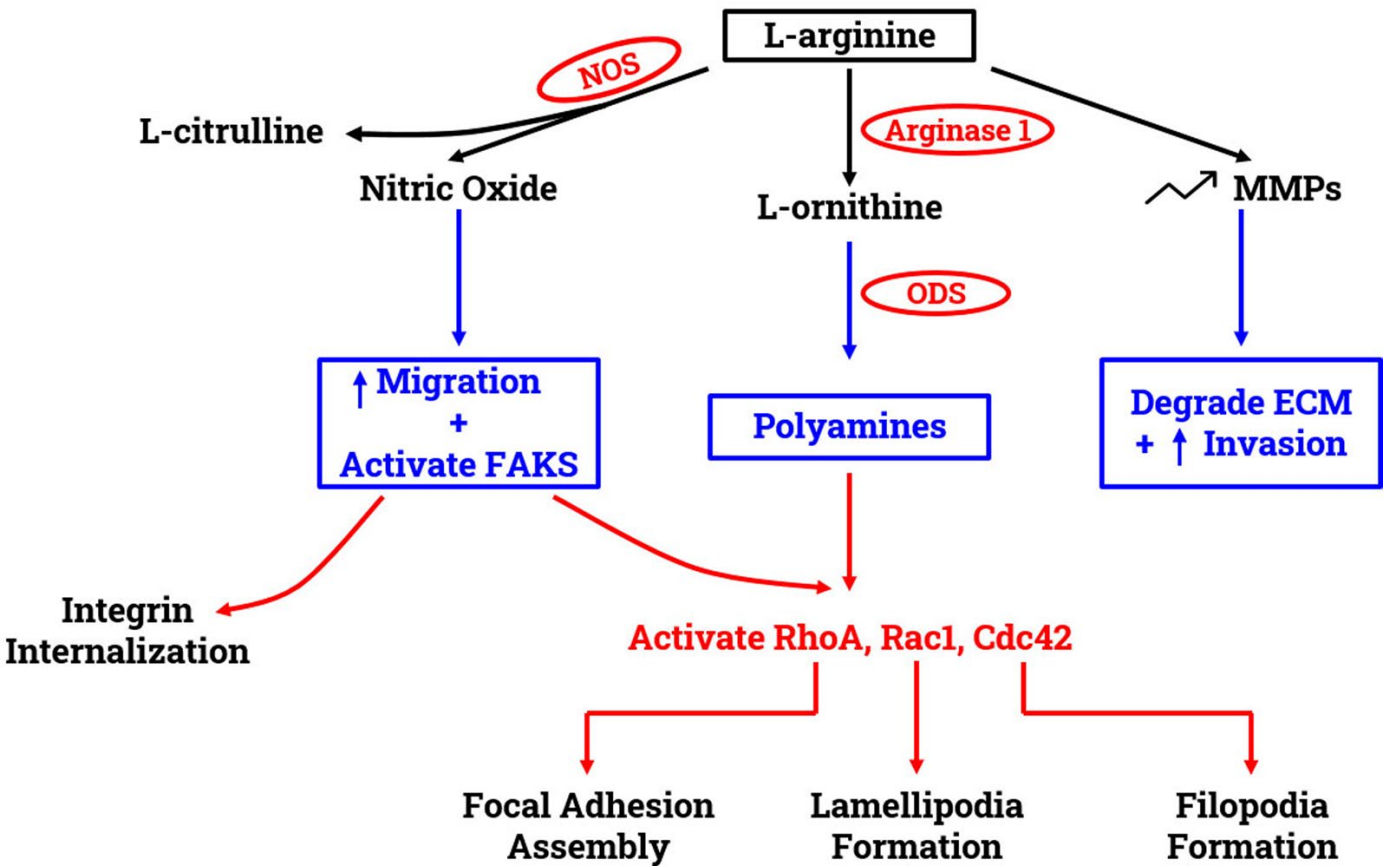
Effect of arginine on migration and invasion. The utilization of l-arginine by NOS produces l-citrulline and nitric oxide. The latter promotes cell migration and activates the focal adhesion kinases (FAKS). Activated FAKs induce the internalization of integrins. l-arginine is also metabolized by Arginase 1 to produce l-ornithine with is in turn utilized by ODS to produce polyamines. Polyamines stimulates the activation of the small Rho GTPases RhoA, Rac1 and Cdc42. l-arginine increases the expression of MMPs, which results in an increased matrix degradation and invasion

**Fig. 2:**
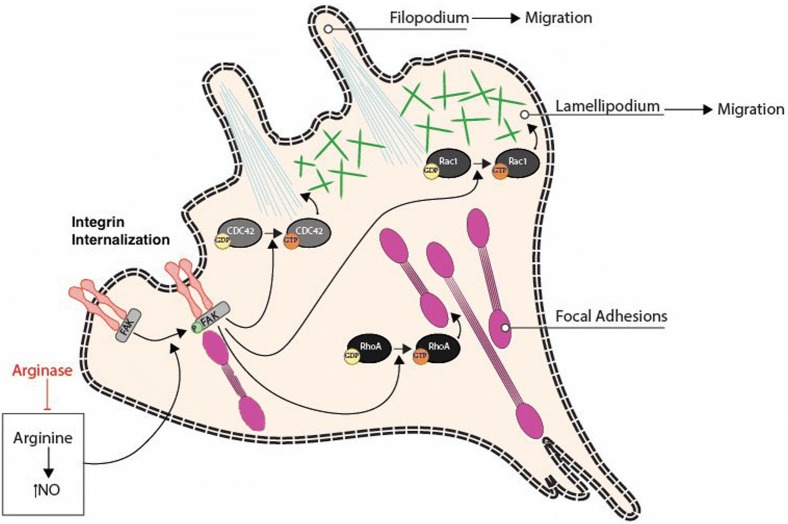
Arginase inhibits cytoskeletal reorganization needed for cell migration. Upon the production of nitric oxide from l-arginine, FAK gets phosphorylated activating Cdc42, Rac1 and RhoA by replacing GDP with GTP. Collectively, RhoGTPases lead to the regulation of the actin cytoskeleton and the formation of actin structures and dynamics needed for cell migration, such as filopodia and lamellipodia formation and focal adhesion dynamics. These are required for cancer cell migration, hence arginase leads to the inhibition of cancer cell migration through the inhibition of these events

Arginine dependent migration requires arginine to be metabolized by two major enzymes, arginase 1 (Arg1) and nitric oxide synthase (NOS). The latter metabolizes arginine to NO and citrulline [33]. During the process of wound healing, the levels of NO elevate, promoting cell migration and proliferation [34, 35]. In case of arginine dependent cell migration, l-arginine is converted to l-citrulline and NO. This NO activates Focal adhesion kinase (FAK) signaling cascade, which internalizes integrin and regulates focal adhesion (FA) assembly and disassembly (Figs. 1 and 2). Thus, when arginine was depleted from the environment using HuArgI(Co)-PEG5000, the wound healing assay results showed a decrease in the colorectal cancer cells’ migration capability. However, when l-citrulline was added with the treatment, the rate of cell migration was restored [29]. Moreover, in another study, normal intestinal cells were supplied with NOS inhibitors, which resulted in a decrease in cell migration. However, when NOS was added in the presence of arginine and NO secondary messenger in post-injury intestinal epithelial cells, cell migration was directly stimulated [36, 37]. This finding was supported by several studies done on other epithelial cell types [37].

Depriving the cell from arginine also plays a role in decreasing the cell’s capability in adhering to the ECM; this was observed in cell adhesion assay done on Caco-2 cells. For the cell to adhere and migrate properly, an interaction between FA molecules and actin cytoskeleton is necessary in the presence of FAK [38]. A study done on human intestinal epithelial cells showed that FAK plays a crucial role in inducing adhesion to the ECM [39]. Moreover, when FAK was inhibited, cell migration got hindered by preventing the newly formed protrusion from getting fixed on the ECM [36]. Immunostaining analysis of FA structure of colorectal cancer cells revealed that the size of FA appears to be smaller after arginine deprivation. Hence, the latter hindered the maturation of FAs, impairing cellular adhesion [29]. Other studies showed that NO and arginine increase FAK phosphorylation with arginine being the main contributor [35, 36].

In addition to NOS, the intracellular Arg1 enzyme also utilizes arginine. It converts arginine to ornithine, which is the precursor of polyamines. One of the enzymes used in polyamine biosynthesis is ornithine decarboxylase (ODC). Polyamine is required during cell migration for forming lamellipodia, stress fibers, and integrin subunit a1. It also increases the K+ channel mediated Ca^2+^ influx and aids FAK in inducing paxillin phosphorylation [32, 39–42]. Targeted inhibition of ODC was correlated with abnormal morphology of actin-cytoskeleton of metastatic cells during migration. Moreover, exogenous supply of polyamine putrescine was able to restore cellular morphology and migration capabilities [36, 43, 44]. A study suggested that Arg1 could be a prognostic biomarker for hepatocellular carcinoma (HCC) patients because higher levels of Arg1 was associated with aggressive tumor growth, higher levels of alanine, aminotransferase, and glutamyltranferase. Overexpression of Arg1 significantly increased cell migration while knocking down Arg1 reduced cell migration when tested on Huh7, liver cell, in vitro [45].

Depriving the cell from arginine also decreases the cells’ capability to break through the ECM and invade. For the cancer cell to remodel the ECM and facilitate invasion, it utilizes matrix metalloproteinases (MMPs), a family of extracellular zinc-dependent proteinases. They can degrade the ECM macromolecules, removing the physical barrier that is stopping the tumor cells from invasion [46, 47]. MMPs are numerous and produced in their inactive state, so they require proteolytic remodeling to become active. These enzymes can adjust signals and physiological processes to establish molecular communication between the tumor cells and the host tissue stroma [47, 48]. Invasion assay results showed that arginine depletion hindered the colorectal cancer cells’ invasive capability. These results were supported by western blots done for MMP2 and MMP9. The blots indicated a significant decrease in MMP expression levels when the cells were treated with HuArgI(Co)-PEG5000 [29] (Fig. 1). Another study done on hepatocellular carcinoma cells Huh7 showed that overexpression and downregulation of Arg1 enhances and hinders the Huh7 invasive capabilities, respectively. The study also introduced the role of Arg1 as a crucial element in promoting cell migration and invasion through enhancing the epithelial-to-mesenchymal transition [45].

Different regulatory signals, at the leading and trailing ends of the cell, work on mediating cell migration [48]. These signals include Rho GTPases, PI3K, integrins and microtubules, whose interlink and positive feedback loops regulate cell polarity [41, 49, 50]. The most significant Rho GTPases are RhoA, Rac1 and Cdc42 that regulate cell adhesion, migration, and invasion. Moreover, they also play an important role in regulating intracellular junctions and cell to ECM interactions [51–55]. When polyamine is depleted, all three Rho-GTPases’ functions (RhoA, Rac1, and Cdc42) are affected, which in turn affects cell migration (Figs. 1 and 2). RhoA assembles focal adhesions, Cdc42 regulates the formation of filopodia, and Rac1 regulates the formation of lamellipodia [51, 53–55]. Arginine depletion hinders the activation of RhoA in colorectal cancer cells which was evident by fluorescence energy transfer (FRET) using a CFP/YPF biosensor [29]. In another study, NO was shown to stimulate Cdc42 and Rac1, which activate Hypoxia-inducible factor 1 (HIF-1) that promotes macrophage migration [56]. Moreover, a study reported that an increase in NO levels cause an increase in pancreatic cancer cells invasiveness through RhoA activation [35, 57] (Fig. 2).

## Conclusion

Many malignant tumors featured high metabolic demand for specific amino acids to meet their rapid growth. Thus, amino acid deprivation is a novel therapy with arginine as a major target. Arginine is known to be a nutritionally essential amino acid with the ability to regulate cell activities and influence cancer cell’s vitality, motility, adhesion, and invasion. Numerous clinical investigations are being held in order to fully grasp the efficiency of arginine deprivation as a therapy to target arginine auxotrophic tumors.

Previous studies showed that arginine is converted by the cell to NO and polyamines that contribute to cell migration. However, the precise role of polyamines and NO are still poorly understood. Hence, further studies should be conducted to enhance our knowledge regarding the complex mechanisms that regulate NO’s and polyamines’ roles in tumor progression. Moreover, more studies should focus on the effect of arginine deprivation on other aspects including the function of peptidylarginine deiminases (PAD). Further studies should be done in order to investigate the relation between arginine deprived, PAD inhibition, and cell death. PAD isotypes are transcriptional co-regulators for various factors, including P53 and tumor growth gene erythropoietin (EPO). It is shown in many types of cancer cells that levels of PAD are elevated, and downregulating PAD hinders cancer cell’s migration and proliferation capabilities [58, 59]. Thus, it would be interesting to study the effect of arginine depletion on PAD’s activation in arginine auxotrophic cancer cells.

## Data Availability

Not applicable.

## Abbreviations

ADI: Arginine deaminase
ASL: Arginosuccinate lyase
ASS1: Arginosuccinate synthase-1
Co: Cobalt
CRC: Colorectal cancer
ECM: Extracellular matrix
EPO: Erythropoietin
FA: Focal adhesions
FAK: Focal adhesion kinases
GBM: Glioblastoma multiforme
HuArgI: Human l-arginase
MMP: Matrix metalloproteinase
NO: Nitric oxide
NOS: Nitric oxide synthase
OCT: Ornithine transcarbamylase
PAD: Peptidylarginine deiminase
PEG: Polyethylene glycol
